# Supplementary figures and images for: Non-canonical amino acid incorporation enables minimally disruptive labeling of stress granule and TDP-43 proteinopathy
Source: eLife. 2026 Jul 3;14:RP109452. doi: 10.7554/eLife.109452 (PMC13331479; doi:10.7554/eLife.109452)

### Source data for Figure 1K

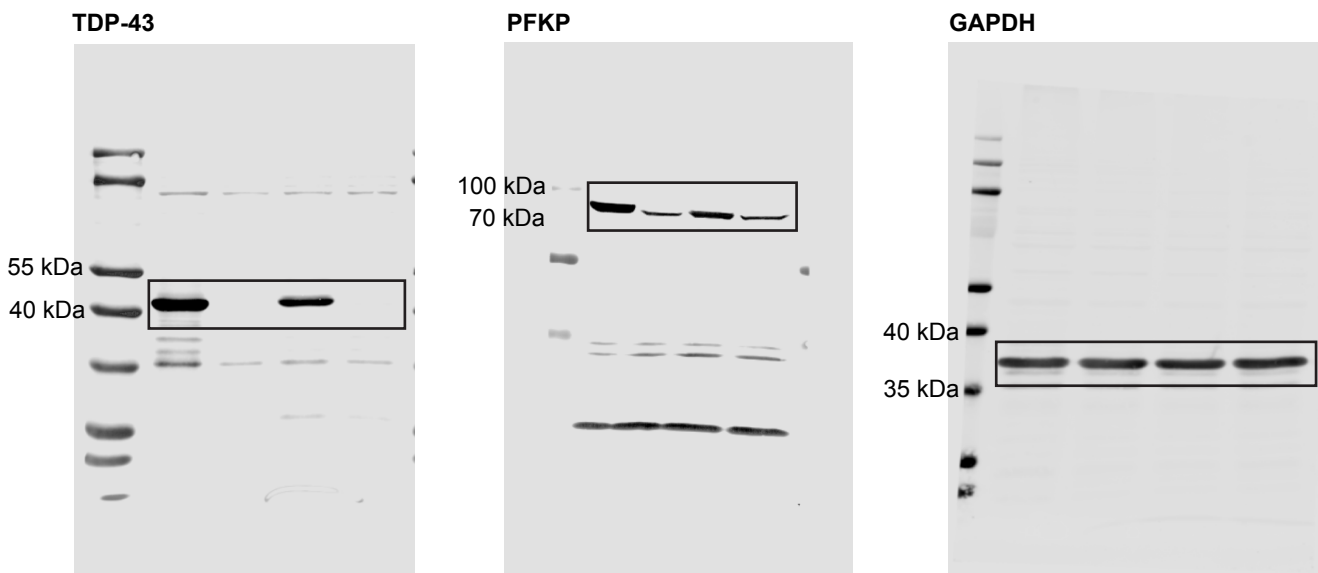

### Source data for Figure 1H

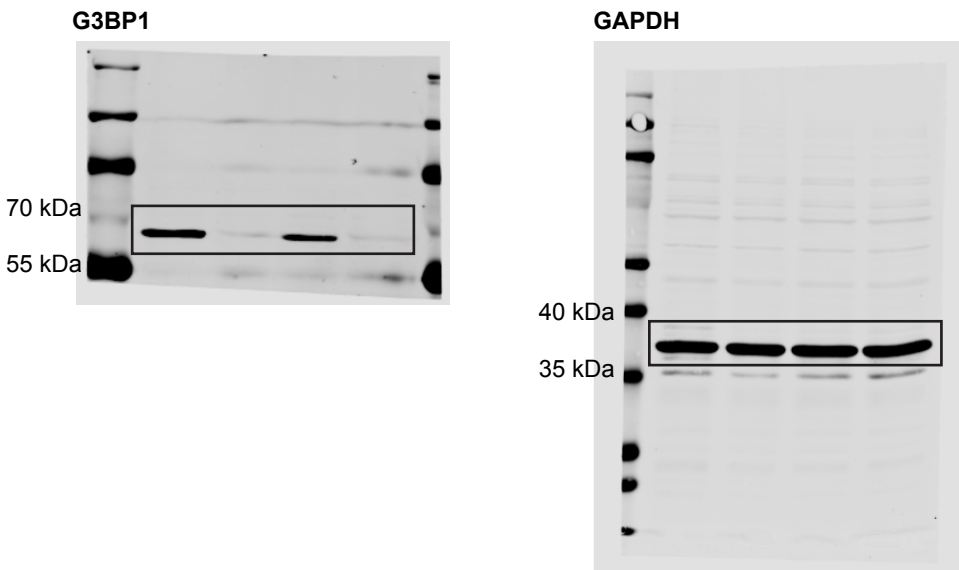

Supplement: Figure 1—source data 2. [file elife-109452-fig1-data2.pdf]

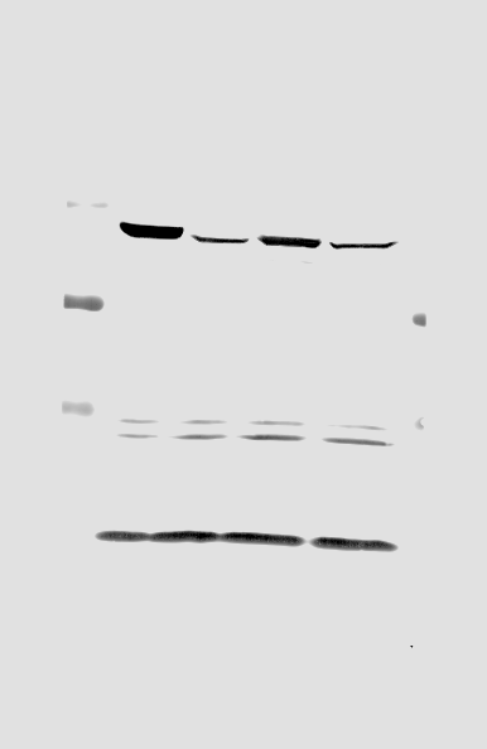

Supplement: Figure 1—source data 3. [file elife-109452-fig1-data3.zip › source data-wb-Figure 1/Fig.1K-PFKP.tif]

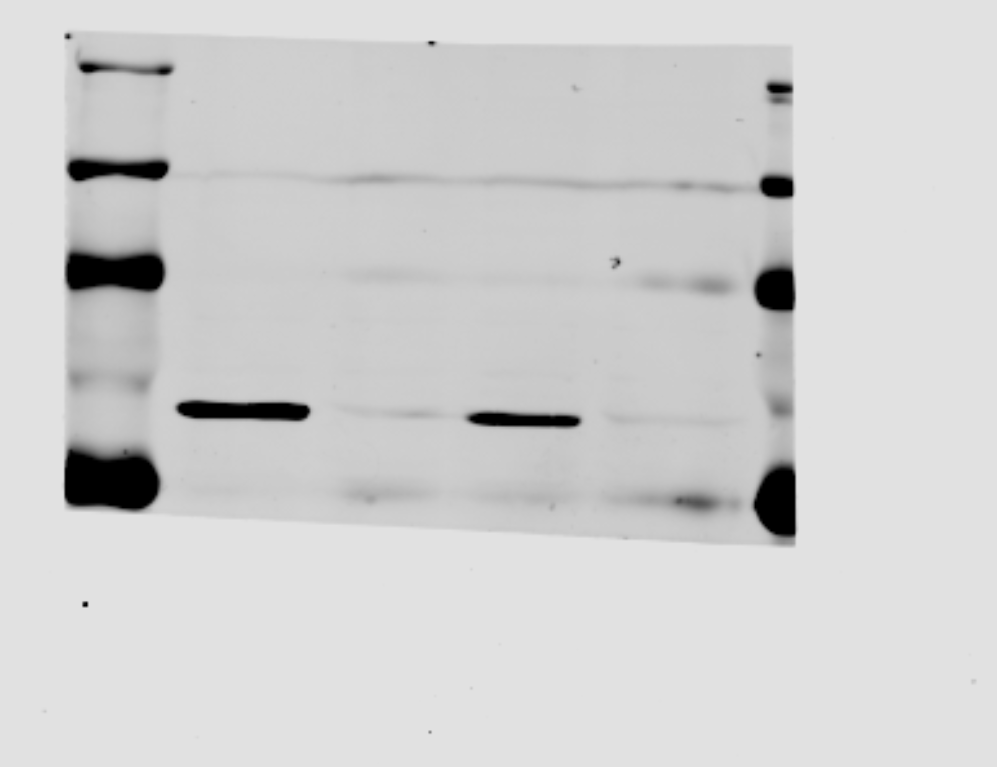

Supplement: Figure 1—source data 3. [file elife-109452-fig1-data3.zip › source data-wb-Figure 1/Fig.1H-G3BP1.tif]

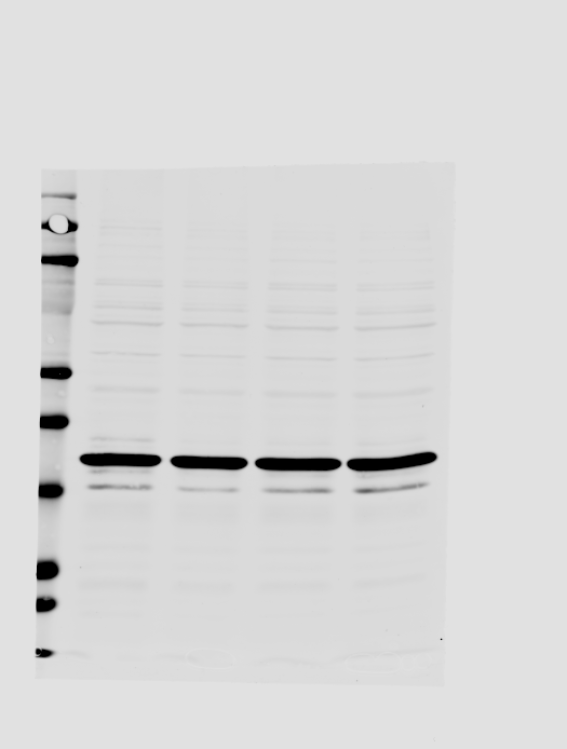

Supplement: Figure 1—source data 3. [file elife-109452-fig1-data3.zip › source data-wb-Figure 1/Fig.1H-GAPDH.tif]

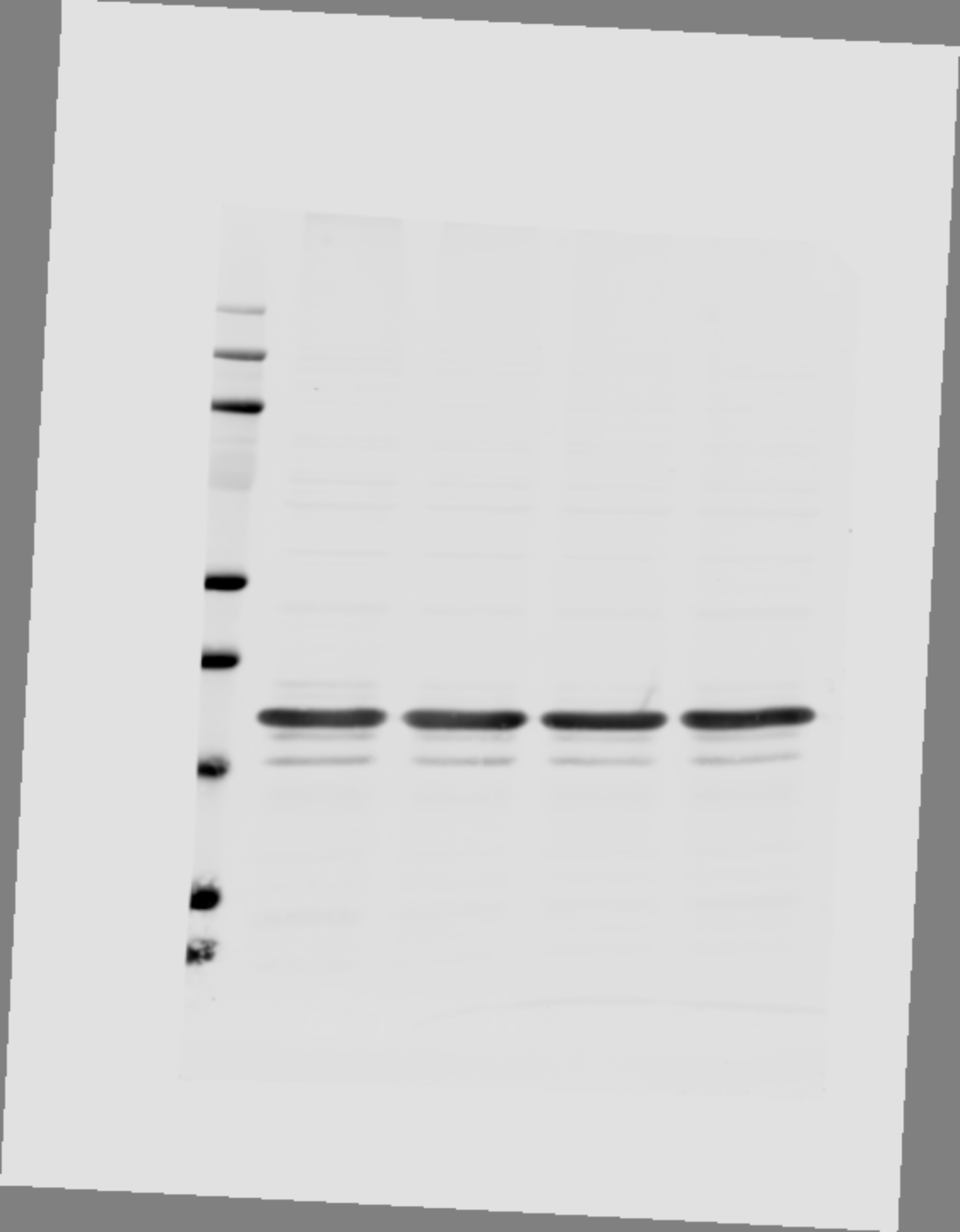

Supplement: Figure 1—source data 3. [file elife-109452-fig1-data3.zip › source data-wb-Figure 1/Fig.1K-GAPDH.tif]

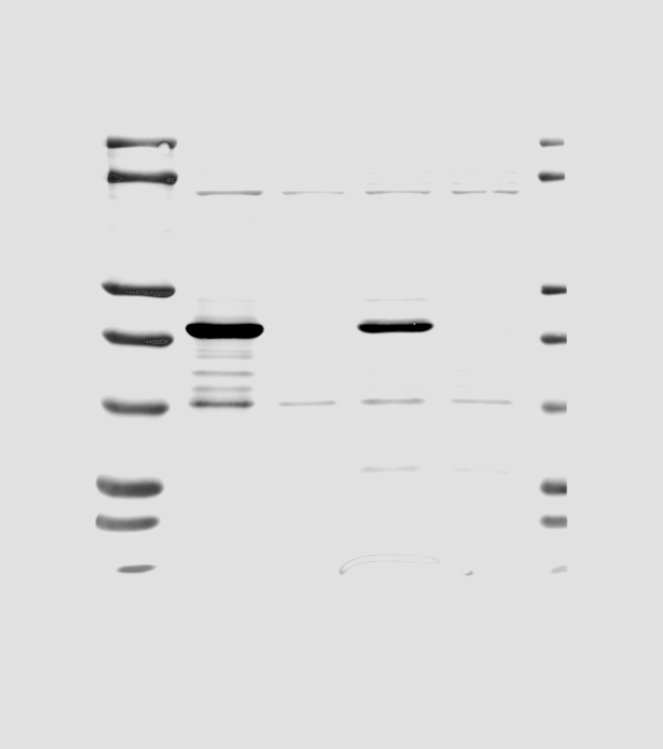

Supplement: Figure 1—source data 3. [file elife-109452-fig1-data3.zip › source data-wb-Figure 1/Fig.1K-TDP-43.tif]
