# Supplementary material for: Non-canonical amino acid incorporation enables minimally disruptive labeling of stress granule and TDP-43 proteinopathy
Source: eLife. 2026 Jul 3;14:RP109452. doi: 10.7554/eLife.109452 (PMC13331479; doi:10.7554/eLife.109452)
Supplement: Peer review history

# Editor report: eLife Assessment

**DOI:** 10.7554/eLife.109452.3.sa0

**Authors:** Hugo J Bellen

**Affiliations:** Baylor College of Medicine United States

**Keywords:** Solid, Useful

This paper demonstrates that a genetic code expansion to tag two amyotrophic lateral sclerosis (ALS) proteins associated with stress granules is **useful** in an experimental context. The data are **solid** and demonstrate the feasibility of using ANAP-fluorescence for live cell imaging.

# Referee report: Reviewer #1 (Public review)

**DOI:** 10.7554/eLife.109452.3.sa1

Summary:

The authors utilize genetic code expansion to tag TDP-43 and G3BP1, and evaluate this protein tagging system (ANAP) compared to antibodies and evaluate protein trafficking and stress granule formation in response to stress with sodium arsenite treatment. They find similar staining to antibodies in HeLa cells, mouse embryonic stem cells and primary mouse cortical neurons. By incorporating the intrinsically fluorescent noncanonical amino acid Anap at carefully selected sites, the authors enable live-cell and neuronal visualization of protein localization, stress-induced redistribution, and dynamic behavior without the structural and functional compromises often associated with large fluorescent protein tags. The work provides technical framework that will be useful for live imaging of tagged proteins.

Strengths:

A key strength is the demonstration of the specificity of the Anap fluorescence signal through appropriate controls and the agreement between Anap labeling and antibody-based detection across multiple cell types, including primary neurons. The ability to visualize stress-induced redistribution of both G3BP1 and TDP 43 in living cells highlights the practical value of this approach.

The functional validation of TDP 43-Anap is compelling. The rescue of both cell viability and RNA splicing defects in TDP 43 knockout models provides evidence that Anap incorporation preserves core protein functions. This is important, as functional disruption is a central concern for any alternative tagging strategy applied to aggregation-prone or RNA-binding proteins.

Weaknesses:

While some inherent limitations of genetic code expansion remain (e.g., variable amber suppression efficiency and the inability to directly assess endogenous protein behavior), these are acknowledged and discussed appropriately. Importantly, these limitations do not undermine the central contributions of the study.

# Referee report: Reviewer #2 (Public review)

**DOI:** 10.7554/eLife.109452.3.sa2

In this manuscript, Chen and colleagues describe a novel means of labeling two RNA binding proteins, G3BP1 and TDP-43, using genetic code expansion. Overexpressed constructs that incorporate the intrinsically-fluorescent non-canonical amino acid Anap redistribute to cytoplasmic granules upon application of external stressors such as sodium arsenite. Similar labeling and redistribution of overexpressed G3BP1 and TDP-43 was observed in cultures of mouse primary neurons.

Genetic code expansion and non-canonical amino acid labeling have many advantages over traditional fusion proteins for tracking protein redistribution in living cells. The authors show that they are able to label exogenous G3BP1 and TDP-43 with the non-canonical amino acid Anap, and follow labeled proteins in living cells with and without stress.

I suspect that this method could be incredibly valuable to many investigators studying the dynamics and interactions of proteins that are difficult to label or detect by conventional methods.

Comment on revised version:

The revised manuscript is significantly improved, with added controls and experiments to confirm expression and Anap labeling of G3BP1 and TDP-43.

# Author comment: Author response

**DOI:** 10.7554/eLife.109452.3.sa3

**Authors:** Hao Chen, Haocheng Wang, Yu-Ning Lu, Peng Chen, Zhongfan Zheng, Tao Zhang, Jiou Wang

**Affiliations:** Johns Hopkins University Baltimore United States; Johns Hopkins University Baltimore United States; Johns Hopkins University Baltimore United States; Johns Hopkins University Baltimore United States; Johns Hopkins University Baltimore United States; Johns Hopkins University Baltimore United States; Johns Hopkins University Baltimore United States

The following is the authors’ response to the original reviews.

**eLife Assessment**

Amyotrophic lateral sclerosis (ALS) affects nerve cells in the brain and spinal cord. The authors' approach to use genetic code expansion to tag two ALS proteins associated with stress granules has value and should be useful in the ALS field. Parts of the work are well done, but there are concerns that the evidence is incomplete overall, and additional controls would strengthen the study.

We thank the editors and reviewers for their thoughtful assessment and for highlighting the potential value of applying genetic code expansion (GCE) to study ALSassociated proteins involved in stress granule biology. Our goal in this work was to establish and validate a minimally perturbative labeling strategy using the noncanonical amino acid Anap to monitor the localization and stress-dependent behavior of TDP-43 and G3BP1.

We agree that additional controls can further strengthen the conclusions. In the revised manuscript, we have clarified the experimental design and added essential controls to better support the reliability of the Anap labeling approach (Supplementary Fig. 1).

**Public Reviews:**

**Reviewer #1 (Public review):**

Summary:

The authors utilize genetic code expansion to tag TDP-43 and G3BP1, and evaluate this protein tagging system (ANAP) compared to antibodies, and evaluate protein trafficking and stress granule formation in response to stress with sodium arsenite treatment. They find similar staining to antibodies in HeLa cells, mouse embryonic stem cells, and primary mouse cortical neurons. This is a useful study that demonstrates the utility of ANAP tagging to evaluate ALS proteins.

We sincerely thank the reviewer for the positive assessment of our work and for recognizing the utility of the Anap-based GCE system for studying ALS-associated proteins.

Strengths:

Rescue of cell survival by ANAP-tagged TDP-43 is compelling

We appreciate the reviewer’s highlighting of this point. Demonstrating that TDP43-Anap can rescue cell survival was an important validation in our study, as it indicates that incorporation of the noncanonical amino acid does not substantially disrupt the biological function of TDP-43. Additionally, we also tested the RNA splicing function recovery potency of TDP-43-Anap. As shown in Fig. 1K and 1L, a recovery of expression of PFKP, a protein undergoing cryptic exon when TDP-43 lost its function [1], was observed when expressing TDP-43-Anap in TDP-43 knockout Hela cells.

Weaknesses:

While the ANAP-tagged proteins had similar distributions to antibody staining, there were some discrepancies that may be more explained by the technique than by novel findings, as the authors suggested. The inclusion of additional controls to evaluate this would be helpful.

This is a helpful suggestion. To ensure that the fluorescence signal observed in our experiments was specifically derived from site-specific Anap incorporation rather than background fluorescence, we performed three control conditions. Specifically, we tested: (1) cells cultured with Anap supplement, (2) cells expressing the Anap incorporation system with the addition of Anap, and (3) cells expressing both the TAG-mutated protein plasmid and the Anap incorporation system but without the addition of Anap. These control experiments were performed for both TDP-43 and G3BP1, and no observable fluorescence signal was detected under any of these conditions (Supplementary Fig. 1). We have clarified this control experiment in the revised manuscript.

**Reviewer #2 (Public review):**

Summary:

In this manuscript, Chen and colleagues describe a novel means of labeling two RNAbinding proteins, G3BP1 and TDP-43, using genetic code expansion. Overexpressed constructs that incorporate the intrinsically fluorescent non-canonical amino acid Anap redistribute to cytoplasmic granules upon application of external stressors such as sodium arsenite. Similar labeling and redistribution of overexpressed G3BP1 and TDP43 were observed in cultures of mouse primary neurons.

We are grateful for the reviewer’s accurate summary of our study and recognition of the value of GCE strategy for labeling the RNA-binding proteins G3BP1 and TDP-43.

Strengths:

Genetic code expansion and non-canonical amino acid labeling have quite a few advantages over traditional fusion proteins for tracking protein redistribution in living cells. The authors show that they are able to label exogenous G3BP1 and TDP-43 with the non-canonical amino acid Anap and follow labeled proteins in living cells with and without stress.

We acknowledge the reviewer’s comment on the advantages of GCE-based noncanonical amino acid labeling for studying protein dynamics in living cells.

Weaknesses:

The authors do not convincingly leverage the advantages of genetic code expansion in the current study. There is no specific question posed by the authors that can be or is answered using this approach, and several of the experiments lack critical controls. This is also not the first example of TDP-43 labeling by genetic code expansion (see PMID: 38290242). As a result, the study as a whole adds little to our understanding of protein trafficking and behavior under stress.

We thank the reviewer for raising these important points. Although as reviewer mentioned, genetic code expansion has previously been applied to TDP-43 [2], it mainly employed the photocaged lysine incorporation system to optogenetic control of TDP-43 translocation, and the protein was still labeled by mRubby. Our paper has totally different goal, to establish and validate a minimally perturbative labeling strategy using the intrinsically fluorescent noncanonical amino acid Anap to monitor the localization and stress-dependent behavior of both TDP-43 and G3BP1. And our work extends this approach in several important ways.

First, we demonstrate that Anap incorporation enables visualization of stress-dependent redistribution of both TDP-43 and G3BP1, two key proteins involved in stress granule biology. Importantly, we validate this approach across multiple cellular systems, including HeLa cells, mouse embryonic stem cells, and primary mouse cortical neurons, which broadens the applicability of this labeling strategy.

Second, we provide functional validation of the Anap-tagged protein, showing that TDP43-Anap rescues both cell survival and RNA splicing activity in TDP-43 knockout cells, including restoration of PFKP expression, a known cryptic exon target of TDP-43. These results support that Anap incorporation does not substantially disrupt protein function.

We performed additional control experiments to ensure the specificity of the labeling system. Specifically, we tested three control conditions: (1) cells cultured with Anap supplement, (2) cells expressing the Anap incorporation system with the addition of Anap, and (3) cells expressing both the TAG-mutated protein plasmid and the Anap incorporation system but without the addition of Anap. These control experiments were performed for both TDP-43 and G3BP1, and no observable fluorescence signal was detected under any of these conditions (Supplementary Fig. 1).

We agree that the manuscript would benefit from clearer articulation of the advantages of genetic code expansion in this context. Accordingly, we have revised the manuscript to more explicitly emphasize how Anap labeling provides a minimally perturbative alternative to large fluorescent protein fusions, which can alter the phase behavior and localization of stress granule proteins.

“Conventional fluorescent protein tags have enabled visualization of TDP-43 and G3BP1 in living cells; however, these approaches can perturb the native biophysical properties of the proteins being studied. For example, GFP or other fluorescently tagged TDP-43 usually requires additional modifications, such as deletion of the nuclear localization signal (NLS) [3, 4], to induce cytoplasmic inclusion formation. Such manipulations introduce non-physiological conditions that may alter the native trafficking and aggregation behavior of TDP-43. As for G3BP1, tags like GFP may also cause unexpected effects on the phase separation or other dynamics of the protein. In contrast, Anap based GCE strategy allows the minimally perturbative labeling and visualization of protein localization and stress-induced redistribution while preserving native protein architecture and function of both proteins. Importantly, the approach provides a generalizable genetically encoded platform for quantitatively examining the behavior of ALS-associated proteins in living cells. By enabling faithful monitoring of protein trafficking and stressgranule dynamics without extensive protein engineering, Anap-based GCE can offer a powerful strategy for probing molecular-scale mechanisms underlying ALS-linked proteinopathies”.

**Recommendations for the authors:**

**Reviewer #1 (Recommendations for the authors):**

(1) Figure 1A

The authors report that the nuclear staining of G3BP1 by ANAP labeling shows the presence of nuclear pools of G3BP1 that aren't detected with antibody staining. However, unspecific nuclear staining by aminoacylated tRNAs bound to synthetases has been described. It would be important to have a control to evaluate for this possibility.

This is an important point. We agree that the nuclear ANAP signal should be carefully controlled to exclude the possibility of nonspecific staining arising from the Anap incorporation machinery itself, such as aminoacylated tRNAs and/or synthetases.

To address this concern, in methods and material part, we note that after DPBS washes to remove excess Anap, cells were incubated in fresh medium for 2 hours to allow sufficient time for the decay of unstable aminoacylated tRNAs, which are generally cleared within minutes to tens of munites [5].

Also, we performed three control conditions for both TDP-43 and G3BP1: (1) cells cultured with Anap supplement, (2) cells expressing the Anap incorporation system with the addition of Anap, and (3) cells expressing both the TAG-mutated protein plasmid and the Anap incorporation system but without the addition of Anap. Under all three conditions, we observed no detectable fluorescence signal (Supplementary Fig. 1).

In addition, as shown in Fig. 1I, the nuclear signal of G3BP1-Anap partially colocalizes with the nuclear signal of TIA-1 in several condensate-like structures. This observation further supports that the nuclear Anap signal reflects protein-associated localization rather than nonspecific fluorescence, as it overlaps with a known RNA-binding protein that can form nuclear condensates under certain conditions.

(2) Figure 1A, 1B

Anap labeling appears to stain fewer cytoplasmic structures compared to antibody staining for both G3BP1 and TDP-43 after sodium arsenite treatment. Quantification would be useful to address whether this is the case. If so, might this be due to unincorporated/truncated proteins competing with Anap-labeled proteins?

We appreciate the reviewer’s helpful suggestion. To address this point, we performed quantitative colocalization analysis using Fiji/ImageJ, calculating the Pearson correlation coefficient (R) for regions of interest between the Anap signal and antibody staining. These analyses indicate a strong overall agreement between the two detection methods under stress conditions, supporting that Anap labeling reliably reports the localization of both G3BP1 and TDP-43 (see Fig1. A, B).

Regarding the possibility that truncated or unincorporated proteins could influence the observed signal, we note that fluorescence from Anap depends on successful amber suppression and incorporation of Anap at the engineered TAG site. Proteins that fail to incorporate Anap, such as truncated products generated by premature termination, would not produce fluorescence, and therefore would not contribute to the Anap signal. Thus, the Anap fluorescence selectively reports the population of successfully labeled full-length proteins, whereas antibody staining detects both labeled and unlabeled protein pools. This difference may partially explain why antibody staining appears to label a larger number of cytoplasmic structures.

(3) Figure 1F

FRAP of G3BP1-GFP in stress granules is slower than in previous publications. The underlying reasons for this should also be addressed.

We thank the reviewer for this important observation. Differences in FRAP recovery kinetics of G3BP1 in stress granules may arise from several experimental variables that are known to influence stress granule dynamics. These include differences in cell type, expression levels of G3BP1-GFP, and imaging or photobleaching parameters. In our experiments, FRAP measurements were performed under specific conditions optimized for our experimental system, which may lead to recovery kinetics that differ from those reported in previous studies.

(4) Figure 1H

A full-size Western blot would be useful to evaluate for amount of truncated protein for G3BP1 and TDP-43. Could truncated proteins be competing with and altering ANAPtagged G3BP1 and TDP-43 localization in response to stress? This should be addressed.

We acknowledge this important point. Full-size Western blotting can provide information on the overall presence of truncated species in the transfected population; however, it represents a bulk measurement and does not capture cell-to-cell variability in amber suppression efficiency at the single-cell level. We therefore cannot exclude the possibility that truncated products are present at varying levels in individual cells and may contribute, directly or indirectly, to differences between antibody staining and Anap fluorescence.

Importantly, we observe that cells with successful Anap incorporation consistently exhibit strong antibody staining for TDP-43 or G3BP1, indicating that full-length protein is the predominant species in these cells. Because Anap fluorescence depends on successful amber suppression, it selectively reports the full-length protein population, whereas truncated products are not detected in the imaging assay. The concordance between Anap fluorescence and antibody staining therefore argues against a major contribution of truncated species to the observed localization patterns (Supplementary Fig. 1).

Accordingly, we interpret the Anap signal as reflecting the localization of successfully labeled full-length protein, while acknowledging that heterogeneity in suppression efficiency is an important limitation of the current approach.

(5) Figure 3

This is a well-designed diagram.

We are grateful for the reviewer’s positive feedback on the diagram and are pleased that the schematic effectively illustrates the experimental design and the principles of the genetic code expansion strategy used in this study.

**Reviewer #2 (Recommendations for the authors):**

The authors present a one-sided viewpoint concerning the connection between stress granules and disease (lines 45-46). A more balanced discussion is recommended, including data arguing against a role for abnormal stress granules in neurodegeneration.

This is an important suggestion. We agree that the relationship between stress granules and neurodegeneration remains an active area of investigation and that evidence both supporting and questioning a causal role of stress granules in disease has been reported. In the revised manuscript, we have modified the Introduction to provide a more balanced discussion of this topic.

“Altered stress-granule dynamics have been associated with ALS/FTD [6, 7]; however, whether stress granules directly drive neurodegeneration remains debated, as several studies suggest that stress granules primarily function as protective stress responses [8].”

(1) A central rationale for the study is missing. The authors state only that G3BP1 and TDP-43 'undergo dynamic stress-dependent redistribution, making them ideal candidates for minimally invasive, site-specific fluorescent labeling.' Is there a controversy or question that can be resolved using these approaches?

We thank the reviewer for raising this important point. The central motivation of this study is that the dynamic behavior and phase separation properties of stressgranule proteins are highly sensitive to protein modifications and tagging strategies.

“Conventional fluorescent protein tags have enabled visualization of TDP-43 and G3BP1 in living cells; however, these approaches can perturb the native biophysical properties of the proteins being studied. For example, GFP or other fluorescently tagged TDP-43 usually requires additional modifications, such as deletion of the nuclear localization signal (NLS) [3, 4], to induce cytoplasmic inclusion formation. Such manipulations introduce non-physiological conditions that may alter the native trafficking and aggregation behavior of TDP-43. As for G3BP1, tags like GFP may also cause unexpected effects on the phase separation or other dynamics of the protein.”

(2) Related to this, there is little context for how or why genetic code expansion is utilized for these studies

We agree that the rationale for using genetic code expansion should be more clearly explained. In this study, genetic code expansion was employed to enable sitespecific incorporation of the small fluorescent noncanonical amino acid Anap, allowing minimally perturbative labeling of proteins of interest.

“Anap based GCE strategy allows the minimally perturbative labeling and visualization of protein localization and stress-induced redistribution while preserving native protein architecture and function of both proteins. Importantly, the approach provides a generalizable genetically encoded platform for quantitatively examining the behavior of ALS-associated proteins in living cells. By enabling faithful monitoring of protein trafficking and stress-granule dynamics without extensive protein engineering, Anapbased GCE can offer a powerful strategy for probing molecular-scale mechanisms underlying ALS-linked proteinopathies.”

(3) The justification for the criteria for selecting the site for incorporation of non-canonical amino acids in G3BP1 or TDP-43 is missing.

We acknowledge this important comment and agree that the rationale for selecting the incorporation sites should be stated more clearly.

“For TDP-43, the incorporation site was selected to avoid the major functional domains involved in RNA binding, nuclear localization, and aggregation-related behavior, thereby reducing the likelihood that Anap incorporation would perturb its native trafficking or function. For G3BP1, the selected site was chosen to minimize interference with domains important for stress granule assembly, RNA binding, and protein-protein interactions. More generally, we aimed to place the ncAA at positions likely to be solventaccessible and tolerant of substitution, while avoiding highly conserved or functionally essential residues.”

(4) Studies in Figures 1 and 2 lack essential controls, including background signal from Anap in non-transfected cells, or those transfected with plasmids lacking the tRNA or tRS.

This is an important point, also raised by Reviewer 1. To evaluate potential background fluorescence arising from Anap or the labeling system, we performed several control experiments. Specifically, we examined three conditions: (1) cells cultured with Anap supplement, (2) cells expressing the Anap incorporation system with the addition of Anap, and (3) cells expressing both the TAG-mutated protein plasmid and the Anap incorporation system but without the addition of Anap. Under all three conditions, we observed no detectable fluorescence signal (Supplementary Fig. 1).

(5) Another marker of stress granules should be used for confirming the identity of G3BP1-Anap (+) or TDP-43-Anap (+) structures, including TIA1, TAF15, or polyA RNA.

We appreciate this helpful suggestion. To further confirm the identity of the stress granule structures observed in our experiments, we performed colocalization analysis with TIA-1, a well-established marker of stress granules. The results have been included in revised manuscript.

“Additionally, we examined the colocalization of G3BP1-Anap with TIA-1, another established stress granule marker. Under stress conditions, G3BP1-Anap largely colocalized with TIA-1 within stress granules. Interestingly, under basal conditions, the nuclear signal of G3BP1-Anap, which was not detected by antibody staining, appeared to partially colocalize with TIA-1 in several condensate-like structures. (Fig. 1I).”

(6) There is no information on the number of granules bleached or the number of cells selected for FRAP studies. There is no information on the shaded areas in Figure 1F or 1G, and no information on statistical comparisons between regressions in Figure 1F.

We thank the reviewer for pointing out these omissions. We have revised the figure legends to clarify these details.

“One granule from each of three independent cells was selected and photobleached for FRAP analysis.”

“Here, error bars with filled area are used for better data presentation. FRAP recovery curves were compared using two-way ANOVA.”

(7) Protein dynamics measured by FRAP are highly dependent on the concentration and/or expression level of each protein. Because of this, the authors need to control for expression level in all FRAP studies.

We agree that protein concentration and expression level can influence FRAP recovery kinetics. Since Anap incorporation is based on amber suppression, and the suppression rate in each cell varies, so it is difficult to control the expression of Anap labeled proteins, however, to minimize this potential effect, we performed FRAP measurements on cells exhibiting comparable fluorescence intensities, which served as a proxy for similar expression levels of the labeled proteins. In addition, FRAP analyses were conducted on individual granules within cells expressing moderate levels of the protein, avoiding cells with unusually high fluorescence intensity that might reflect overexpression.

Furthermore, fluorescence recovery was normalized to the pre-bleach intensity of the selected granules, which reduces variability arising from differences in overall expression levels between cells.

(8) There is no point of reference for TDP-43-Anap FRAP results in Figure 1G. Additional studies using variants harboring a mutated NLS (mNLS) can be used in place of TDP43-YFP.

This is a helpful suggestion. In response, we have performed additional FRAP experiments using TDP-43^ΔNLS^, a commonly used construct that promotes cytoplasmic localization and facilitates analysis of TDP-43 granules. The results from TDP-43^ΔNLS^ have now been included as a reference for the FRAP measurements of TDP-43-Anap in the revised manuscript (Fig. 1D, 1G).

“We then used YFP-tagged nuclear localization signal (NLS)-deleted TDP-43 (TDP43^ΔNLS^-YFP) as a reference and performed FRAP analysis to compare the mobility of TDP-43-Anap and TDP-43^ΔNLS^-YFP. Fluorescence recovery of TDP-43-Anap reached ~45% within 20 s after photobleaching, consistent with liquid-like dynamics. In contrast, TDP-43^ΔNLS^-YFP showed only ~22% recovery, suggesting more solid-like dynamics (Fig. 1D, 1G). These results are consistent with previous reports describing relatively immobile aggregates formed by TDP-43^ΔNLS4^and illustrate the advantage of Anap-based labeling, which preserves native protein properties and enables real-time assessment of protein dynamics without introducing disruptive mutations.”

(9) There is no point of reference for comparing FRAP results from G3BP1-GFP to G3BP1-Anap. What is the 'gold standard'? Without this, it is difficult to conclude that "... Anap labeling better preserved the native mobility and biophysical properties of G3BP1 than the conventional GFP tag."

We acknowledge this important point and agree that there is currently no definitive gold standard for measuring the native mobility of endogenous G3BP1 within stress granules in living cells. Our intention was not to claim that the Anap-labeled protein definitively represents the native state, but rather to compare the relative effects of different labeling strategies.

Thus, we rewrite the sentence as “These results suggest that G3BP1-Anap displays higher mobility compared with G3BP1-GFP, indicating that Anap labeling may provide a less perturbative approach for monitoring G3BP1 dynamics.”

(10) The WB in Figure 1H is overexposed, making it difficult to compare expression levels between WT and V100Anap-transfected cells. In addition, there is no similar assay for confirming G3BP1-Anap expression.

Thank you for pointing this out. In the revised manuscript, we have replaced the image with a properly exposed Western blot to allow clearer comparison of protein expression levels.

In addition, we have now included a corresponding western blot analysis to confirm the expression of G3BP1-Anap in G3BP knockout U2OS cell (Fig. 1H). These results verify that the Anap-labeled proteins are expressed at detectable levels and support the interpretation of the imaging and FRAP experiments.

(11) Although survival studies in Figures 1I and J are promising, a more convincing demonstration of functional replacement of TDP-43 would involve an assessment of cryptic exon splicing, comparing WT to TDP-43 KO, V100Stop- and V100Anaptransfected cells.

This is a valuable suggestion.

“We also evaluated TDP-43-dependent RNA splicing activity by examining the expression of PFKP, a well-established target that undergoes cryptic exon inclusion upon loss of TDP-43 function17. As shown in Figures 1K and 1L, expression of TDP-43Anap in TDP-43 knockout HeLa cells restored PFKP expression, indicating that the Anap-labeled protein retains functional RNA splicing activity. These results demonstrate that TDP-43-Anap is capable of functionally compensating for endogenous TDP-43, supporting that the incorporation of Anap does not substantially disrupt the protein’s biological function.”

(12) Tuj1 staining in Figure 2 is inconsistent and often fails to confirm neuronal identity.

We thank the reviewer for this important comment. We acknowledge that Tuj1 staining in Figure 2 is variable and, in some cases, does not clearly delineate neuronal identity. Notably, the reduced Tuj1 signal is primarily observed in neurons that express Anap-labeled proteins under sodium arsenite treatment, which likely reflects the combined effects of transfection-associated stress and oxidative stress on neuronal morphology and cytoskeletal integrity.

In addition, transfection efficiency in primary neurons is inherently low and variable, and cells that successfully express the constructs may represent a more stress-sensitive subpopulation, further contributing to variability in staining quality. Despite optimization efforts, these technical constraints limit the consistency of Tuj1 labeling under these experimental conditions.

(13) Close-up images and correlation scatter plots in Figures 1 and 2 do not add very much information.

We thank the reviewer for this comment. To address the reviewer’s concern, we have revised the figure legends to better clarify the purpose of these panels and how they support the quantitative analysis presented in the manuscript.

For scatter plot, “Colocalization threshold analysis was performed in Fiji/ImageJ to calculate the Pearson correlation coefficient (R) for each region of interest (A, B, I, J). The X- and Y-axes represent the fluorescence intensity values of the red and green channels, respectively. When signals are colocalized, pixels with high intensity in one channel correspond to high intensity in the other, forming a diagonal distribution. In contrast, non-colocalized signals cluster along the axes. A higher R value indicates a greater degree of colocalization. Scale bar, 3 μm.”

Same information was added to figure legend of figure 2.

For the scheme, please see line 412-413 in the revised manuscript.

Reference:

(1) Rothstein, J.D. et al. Sporadic ALS induced pluripotent stem cell derived neurons reveal hallmarks of TDP-43 loss of function. Nature Communications 16, 7092 (2025).

(2) Shadish, J.A. & Lee, J.C. Genetically encoded lysine photocage for spatiotemporal control of TDP-43 nuclear import. Biophys Chem 307, 107191 (2024).

(3) Gasset-Rosa, F. et al. Cytoplasmic TDP-43 De-mixing Independent of Stress Granules Drives Inhibition of Nuclear Import, Loss of Nuclear TDP-43, and Cell Death. Neuron 102, 339–357.e337 (2019).

(4) Yan, X. et al. Intra-condensate demixing of TDP-43 inside stress granules generates pathological aggregates. Cell 188, 4123–4140.e4118 (2025).

(5) Walker, S.E. & Fredrick, K. Preparation and evaluation of acylated tRNAs. Methods 44, 81–86 (2008).

(6) Kassouf, T. et al. Targeting the NEDP1 enzyme to ameliorate ALS phenotypes through stress granule disassembly. Science Advances 9, eabq7585 (2023).

(7) Van Nerom, M. et al. C9orf72-linked arginine-rich dipeptide repeats aggravate pathological phase separation of G3BP1. Proceedings of the National Academy of Sciences 121, e2402847121 (2024).

(8) Wolozin, B. & Ivanov, P. Stress granules and neurodegeneration. Nat Rev Neurosci 20, 649–666 (2019).
